# Supplementary material for: Towards unified AI-driven fracture mechanics: the extended deep energy method (XDEM)
Source: Nat Commun. 2026 Aug 15;17:8492. doi: 10.1038/s41467-026-76748-1 (PMC13484471; doi:10.1038/s41467-026-76748-1)
Supplement: Supplementary file 3 [file 41467_2026_76748_MOESM3_ESM.zip › pic/Appendix/UEL/single_crack_FEM.pdf]

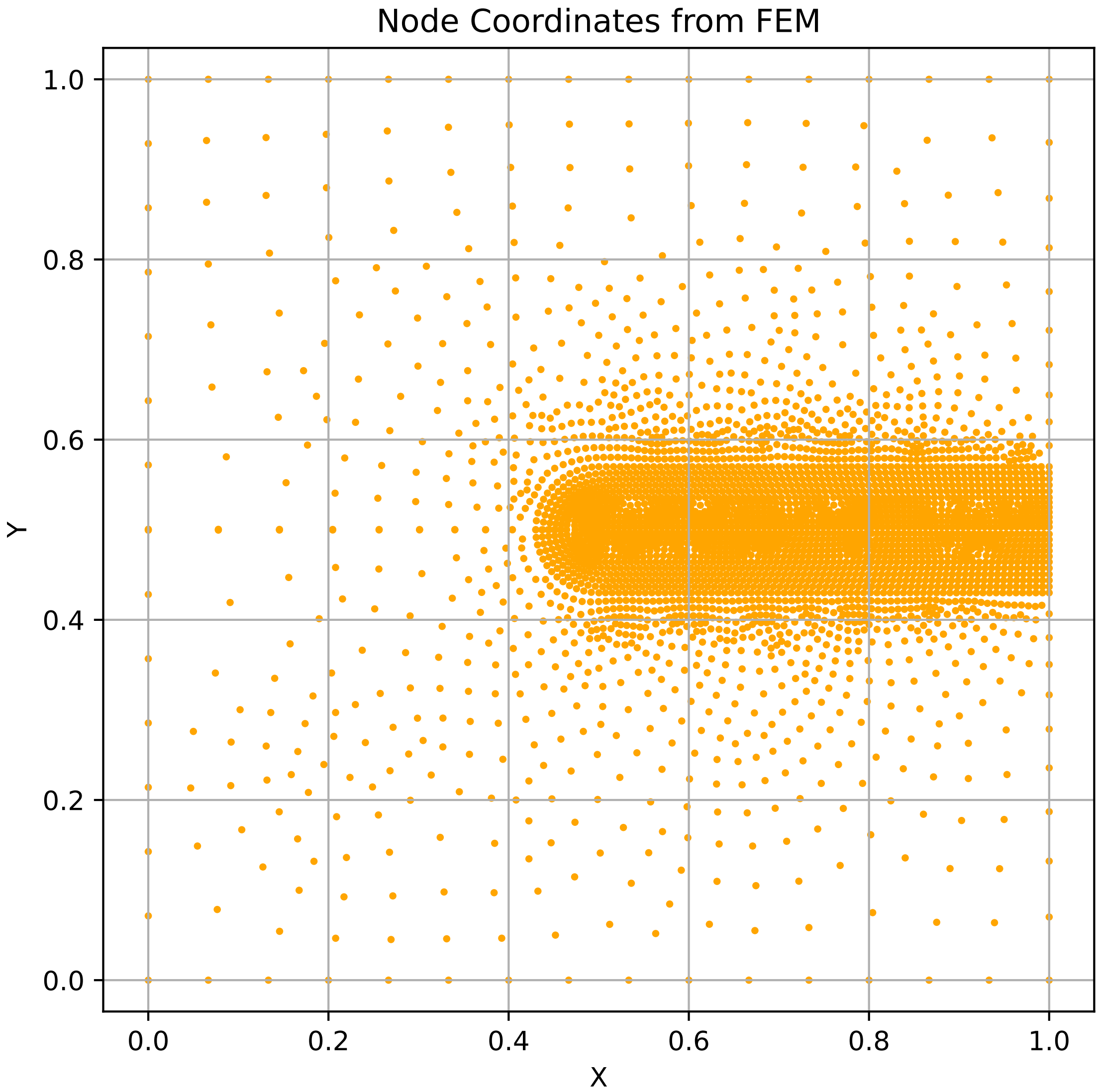
Node Coordinates from FEM
Y
1.0
0.8
0.6
0.4
0.2
0.0
X
0.0
0.2
0.4
0.6
0.8
1.0
